# Headspace Volatile Profile of Fresh-Cut Broccoli Raab in PET Packaging as Affected by Microperforation

**DOI:** 10.3390/foods14244283

**Published:** 2025-12-12

**Authors:** Aysha Saleem, Sandra Pati, Giuseppe Rosiello, Maria Luisa Amodio, Giancarlo Colelli

**Affiliations:** Department of Agricultural, Food, Natural Resources and Engineering Sciences (DAFNE), University of Foggia, Via Napoli 25, 71122 Foggia, Italy; aysha.saleem@unifg.it (A.S.); giuseppe.rosiello@unifg.it (G.R.); marialuisa.amodio@unifg.it (M.L.A.); giancarlo.colelli@unifg.it (G.C.)

**Keywords:** broccoli raab, postharvest factors, microperforation, volatile profile, suphurous compounds

## Abstract

This work investigated the effect of high CO_2_/low O_2_ conditions in polyethylene terephthalate (PET) packaging on the quality of fresh-cut broccoli raab, focusing on volatile organic compounds (VOCs). Fresh broccoli raab was stored for 16 days at 5 °C in microperforated (MP) and in non-microperforated (NMP) PET packaging. During storage, the NMP atmosphere reached approximately 17% CO_2_ and 0% O_2_, while MP packaging maintained approximately 2% CO_2_ and 19% O_2_. NMP samples became unacceptable by day 7 due to loss of firmness and tissue deterioration, while MP samples remained acceptable until day 10, after which yellowing and shrivelling occurred. By day 10, MP VOCs were characterised by ß-caryophyllene, Z-3-hexen-1-ol acetate, and hexen-1-ol, likely generated by enzymatic reactions associated with tissue senescence. NMP packaging showed a high presence of sulphides, isothiocyanates, and nitriles, indicative of severe tissue damage in anaerobic conditions. These alterations in VOCs led to strong, unpleasant sensory notes in NMP samples. This study demonstrated that MP PET packaging can effectively extend the marketable shelf-life of fresh-cut broccoli raab to 10 days, preserving sensory quality and reducing off-odour formation. The findings highlight the potential of microperforated PET as a sustainable solution for enhancing the shelf-life and quality of perishable produce.

## 1. Introduction

Broccoli raab (*Brassica rapa* L.) is a traditional vegetable widely cultivated in Southern Italy [1] and known by regional names such as ‘Cima di rapa’, ‘rapini’, ‘friariello’, and ‘broccoletto’. Its edible part consists of the central inflorescence, including floral heads, auxiliary shoots, and cauline leaves, and is characterised by an aromatic, pungent, and slightly bitter flavour [2]. Its health-promoting properties are primarily attributed to its antioxidant capacity, associated with high levels of vitamins C, E, and A, and a variety of phytochemicals, including flavonoids and glucosinolates [3]. Degradation products of glucosinolates, particularly isothiocyanates and nitriles [4,5], contribute both to the distinctive flavour of the vegetable [6] and to its bioactive potential, with isothiocyanates showing high antimicrobial activity [7] and nitriles providing synergistic effects [8].

Despite these benefits, fresh-cut broccoli raab is not widely commercialised, due to limited consumer familiarity, its regional use and the rapid onset of senescence symptoms, including floret yellowing and off-odours [9,10], often exacerbated by handling and processing.

Minimal processing of broccoli raab offers a valid strategy to provide a convenient, fresh product with extended shelf-life, while ensuring food safety, sensory appeal, and nutritional quality [11]. Optimal harvest maturity and postharvest conditions, including appropriate temperature, relative humidity, and packaging materials, are critical to maintaining product quality [12]. However, studies specifically addressing the impact of the storage behaviour and shelf-life of fresh-cut broccoli raab remain limited.

It is generally recognised that broccoli raab responds positively to modified atmospheres with low O_2_ and high CO_2_ levels under low temperature storage. Nevertheless, atmospheres with O_2_ below 2% and CO_2_ above 10%, and temperature fluctuations, have been shown to negatively affect broccoli raab odour, thereby compromising product quality and shelf life [13]. In related vegetables such as broccoli, cauliflower, and other leafy *Brassica*, several studies have demonstrated that microperforated packaging plays a critical role in regulating volatile organic compounds (VOCs) during storage. Microperforated films maintain more favourable O_2_ and CO_2_ exchange, thereby reducing the accumulation of fermentation-related volatiles and limiting the excessive formation of sulphur-containing compounds typically associated with anaerobic conditions. The number, size, and distribution of microperforations significantly influence the equilibrium atmosphere and, consequently, the metabolic pathways linked to VOC production [14]. Microperforation has been successfully applied to *Brassica* and leafy vegetables such as broccoli [15,16] and pak choi, where it delayed yellowing, preserved bioactives, and maintained flavour. However, research remains limited, and PET-based microperforated films for fresh-cut broccoli raab remain largely unexplored.

Among previously evaluated packaging systems for broccoli raab, microperforated polypropylene/polyamide (PP/PA) films with an initial oxygen concentration of 3–5% allowed for near-optimal equilibrium conditions and extended shelf-life up to 8–12 days. In contrast, polypropylene/polyethylene terephthalate (PP/PET) films led to excessive accumulation of carbon dioxide, resulting in the formation of strong off-odours [17,18]. These undesirable odours have been mainly associated with the production of sulphur-containing volatile compounds, such as dimethyl disulphide (DMDS), methanethiol, dimethyl trisulphide (DMTS), and hydrogen sulphide [19].

Given the growing demand for environmentally sustainable packaging solutions, further investigation into the use of PET-based materials is recommended. Moreover, the current literature on the volatile profile of fresh-cut broccoli raab and its evolution during postharvest storage as a ready-to-use product remains scarce. A recent study by de Chiara et al. [18] analysed VOCs in fresh-cut broccoli raab stored under active modified atmosphere using homogenised samples, thus detecting volatiles primarily originating from enzymatic reactions caused by tissue disruption.

To overcome this limitation, VOCs can instead be analysed directly from the packaging headspace without manipulating the product [20], providing a more realistic representation of the volatiles likely to be perceived by consumers upon package opening. Within this framework, this study aimed to investigate the headspace volatile profile of fresh-cut broccoli raab during refrigerated storage in commercially available PET packaging, comparing microperforated and non-microperforated films. VOCs were determined directly in packaging, without tissue disruption, to support the development of packaging strategies that optimise shelf-life and preserve sensory quality in this highly perishable product.

## 2. Materials and Methods

### 2.1. Chemical Reagents

Extraction solvents (methanol, ethanol) were acquired from Sigma–Aldrich (St. Louis, MO, USA). The SPME fibre holder was obtained from Gerstel (Mülheim an der Ruhr, Germany), and SPME-Fast Fit Fibre Assembly (FFA) divinylbenzene/carboxen/polydimethylsiloxane (DVB/ CAR/PDMS, 50/30 μm film thickness, 1 cm fibre length) was purchased from Supelco (Bellafonte, PA, USA). The commercial analytical standards of all compounds reported in Table 1, with the exception of 3-butenyl isothiocyanate and 2-methyl-butanenitrile, were purchased from Sigma-Aldrich (Milan, Italy) and used for identification purposes.

### 2.2. Broccoli Raab Minimally Processed and Storage

Broccoli raab cv ‘Sessantina’ was provided by local producers in Foggia, harvested at commercial maturity. Ten cm long central parts of the plant with tender leaves and a firm stem were cut using sharp stainless-steel knives. Then, the product was packaged in non-microperforated (NMP) polyethylene terephthalate and in microperforated (MP) polyethylene terephthalate bags with 18 holes (90 µm, inter distance of 2.94 cm) per bag (22 × 16 cm), using a Tecnovac packaging machine (Mod. T520, Grassobbio, BG, Italy) with a 5% O_2_ and 5% CO_2_ active modified atmosphere. Each packaging contained 50 g of broccoli raab and a 350 mL headspace, corresponding to a headspace volume of 7 mL per gram of product. NMP permeability to oxygen (OTR) at 20 °C and 0% RH was 45 mL/m^2^/day, moisture vapour transmission rate (MVTR) at 38 °C and 90% RH was 20 g/m^2^/day; thickness was 30 µm.

A total of 60 bags with fresh-cut broccoli raab were stored for 16 days at a constant temperature of 5 °C. The experimental design consisted of 3 replicates, 2 treatments, and 5 sampling times. Thirty bags were allocated to evaluate gas composition, weight loss, and organoleptic characteristics at 0, 4, 7, 10, and 16 days. The same bags were used sequentially for all three analyses at each time point: first for gas composition, then for weight loss, and finally for sensory evaluation. At each of the 5 sampling times, 6 bags were used, with 3 bags assigned to each of the two treatments (i.e., 3 replicates per treatment per time point). The remaining 30 bags were used to evaluate the volatile profile of packaged broccoli raab at 0, 4, 7, and 10 days. Three replicates per treatment were used at each time point, for a total of 24 bags. By day 16, VOCs were not analysed as both samples were judged unacceptable, and therefore, 6 bags were discarded.

### 2.3. Gas Composition in the Packaging and Weight Loss

At each sampling time, three replicates per treatment were chosen, analysed for gas composition, for volatile profile (see Section 2.5), and then weighted before sensory analysis. Weight loss (WL) was expressed as a percentage of the initial sample weight. O_2_ and CO_2_ levels inside the packaging were tracked using a Dansensor^®^ CheckMate 3 (Dansensor A/S, Ringsted, Denmark) gas analyser. An adhesive rubber septum was used to avoid gas leakage from the packages while inserting the gas analyser probe during measurements from each package.

### 2.4. Visual Appearance and Odour Evaluation

**Figure 1:**
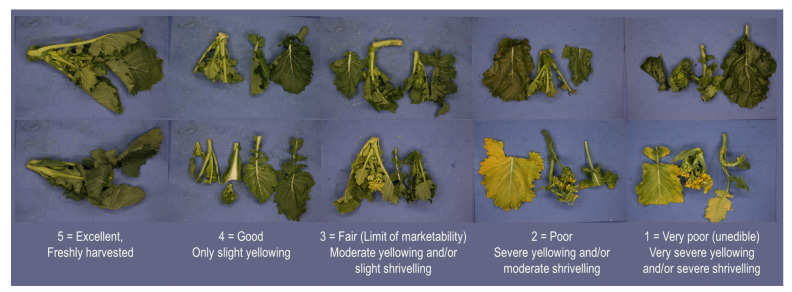
Visual appearance scale used for panel training, calibration, and sensory analysis.

A trained sensory panel of seven researchers from the University of Foggia, all experienced in sensory evaluation, assessed the effects of packaging atmosphere on the visual quality and odour of fresh-cut broccoli raab during storage at 0, 4, 7, 10, and 16 days using 5-point scales [18]. The visual appearance scale, for which panellists were trained using the reference images corresponding to each score shown in Figure 1, was defined as follows: 5 = excellent, no defects; 4 = good, slight yellowing; 3 = fair, moderate yellowing and/or slight shrivelling; 2 = poor, severe yellowing and/or moderate shrivelling; and 1 = very poor, inedible, very severe yellowing and/or severe shrivelling, in case moulds.

The odour scale was defined as: 5 = typical odour, no off-odour, 4 = slight typical odour, no off-odor, 3 = absence of typical odour, 2 = moderate off-odour, 1 = severe off-odour. A score of 3 was considered the threshold of market acceptability. Panel training was carried out twice weekly for 5 weeks. During training, panellists assessed broccoli samples stored under different conditions to represent a range of visual appearances, using the reference images as a guide. For odour assessment, broccoli stored under different conditions was used to provide a range of odour intensities and off-odours. The final four training sessions were dedicated to panel calibration to ensure scoring consistency and agreement among panellists. Although the exact same samples were not used for calibration, broccoli stored under different conditions was again provided to ensure consistent evaluation. Panel performance was assessed with PanelCheck V1.4.2 software. No significant Assessor or Assessor × Product effects were observed, indicating good panel consistency and agreement, while the Product effect was significant (*p* < 0.001), confirming that the panel could reliably discriminate differences between samples.

All samples were coded with random three-digit numbers and presented at room temperature under standard lighting conditions.

### 2.5. Volatile Organic Compounds Extraction and GC-MS Analysis

Volatile organic compounds (VOCs) were extracted by SPME from the packaging before opening at 0, 4, 7, and 10 days. VOCs were extracted directly from the packaging headspace, without any tissue damage, to effectively detect the volatile fingerprint of broccoli raab during storage, without any contribution of the enzymatic products induced by the sample manipulation.

In preliminary work for method development, it was found that headspace sampling for 30 min at 5 °C was the best compromise for the variable ‘time’ to have a good signal response and reduce chemical changes due to the storage time, while maintaining the storage temperature of samples.

A SPME fibre was exposed to the packaging headspace through an adhesive rubber septum for 30 min, at 5 °C, to collect headspace volatiles. As for the initial VOCs, at 0 day, fresh-cut broccoli raab was sampled 1 h after packaging, using the same method. All samples for volatile analysis were run in triplicate. To avoid subsequent contamination of the fibre, blank runs were carried out at the beginning of each sampling day and randomly during the day. The reproducibility of the method was tested by the peak area abundance of a real sample, 1 h after packaging, in six replicate analyses (six different samples, analysed under the same conditions).

SPME samples were inserted into the GC injector port for desorption at 250 °C for 4 min under splitless mode. Analyses were performed using an Agilent 6890 Series gas chromatograph coupled with an Agilent 5975C network mass selective detector (Agilent Technologies, Palo Alto, CA, USA). An INNOWAX capillary column (60 m × 250 µm × 0.25 µm; J&W Scientific Inc., Folsom, CA, USA) was used to separate analytes following a programmed temperature profile at: initial conditions 40 °C for 4 min, then heating up to 140 °C at a rate of 3 °C/min, with a final hold for 10 min. The transfer line was maintained at 280 °C, and helium was used as the carrier gas at a constant flow of 1.0 mL/min. The mass spectrometer operated under electron impact ionisation at 70 eV, with a source temperature of 230 °C, a scan rate of 2.88 scans/s, and a scanning range of 30–400 *m*/*z*. Data acquisition was carried out using MSD ChemStation F.01.01.2317 software (Agilent Technologies), and identification of volatile compounds was based on injections of authentic standards, except for 3-butenyl isothiocyanate and 2-methyl-butanenitrile, which were tentatively identified through spectral comparison with the NIST 02 library (*p* > 80). The semi-quantitative analysis of VOCs was carried out using the integrated peak areas obtained by GC–MS trace. To determine Linear Retention Indices (RIs), a homologous series of straight-chain hydrocarbons (Alkane Standard Solution C8–C20, Sigma Aldrich, Milan, Italy) was analysed under identical conditions.

### 2.6. Statistical Analysis

All measurements are mean ± standard deviation of three replicates per treatment at each storage interval. The data were analysed using one-way ANOVA to find significant differences among treatments during the studied storage period. Tukey’s honest significant difference test (*p* < 0.05) was applied using Statgraphics Centurion XVI version 16.2.04 software. For volatile compounds (peak area values), hierarchical cluster analysis combined with heatmap visualisation was performed using the ClustVis2.0 web tool [21]. The clustering method used for the heatmap was based on correlation for cluster distance for both rows and columns. We applied the average linkage method for both rows and columns to construct the dendrogram. The tree ordering was performed in a way that the tightest clusters were grouped first, allowing for the most related compounds to be clustered together based on their similarity in VOC profiles across storage times.

## 3. Results and Discussion

### 3.1. Gas Composition in the Packaging and Weight Loss

**Figure 2:**
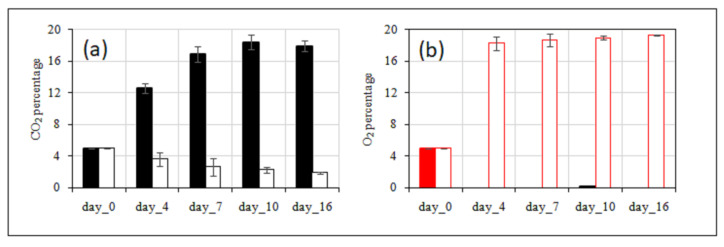
Headspace (**a**), CO_2_, and (**b**), O_2_ percentage in non-microperforated PET (NMP) and in microperforated PET (MP) packaging of fresh-cut broccoli raab stored at 5 °C for 16 days. Values are expressed as mean values of 3 replicates ± standard deviation, shown as vertical bars in the figure. For NMP packaging, the bars are filled with colour, while for microperforated MP packaging, the bars are represented with an empty line (outline).

The evolution of headspace gas composition during storage within packaging is reported in Figure 2. In the NMP packaging, the concentration of CO_2_ increased sharply, reaching 16.9% by day 7, while O_2_ levels declined approaching approximately 0% by day 4, due to the low material permeability and the high respiration rate of the product. These anaerobic conditions remained constant until the end of the storage.

In contrast, the MP packaging exhibited an opposite trend. On day 4, MP maintained CO_2_ at 3.6% and O_2_ at 18.2%; by the end of the storage period (day 16), O_2_ increased to 19.2% and CO_2_ decreased to 1.9%. The high gas permeability of MP enabled a rapid increase of O_2_, which remained nearly constant, almost at air concentration, till the end of the storage period, while CO_2_ levels decreased slowly from 5 to 1.9%, allowing only moderate accumulation.

**Figure 3:**
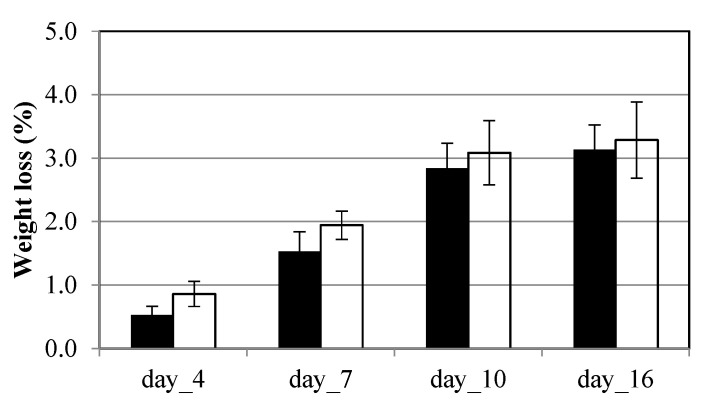
Weight loss, expressed in percentage, of fresh-cut broccoli raab stored in on-microperforated PET (NMP) and in microperforated PET (MP) packaging at 5 °C for 16 days. Values are expressed as mean values of 3 replicates ± standard deviation, shown as vertical bars in the figure. For NMP packaging, the bars are filled, while for microperforated MP packaging, the bars are represented with an empty line (outline).

Results of weight loss are shown in Figure 3. In the case of the MP film, the sample showed higher weight loss due to higher product transpiration; however, the observed differences were not statistically significant. On day 16, samples packaged in NMP exhibited a weight loss of 3.1%, which was not statistically different from the MP sample (3.3%).

### 3.2. Organoleptic Evaluation

**Figure 4:**
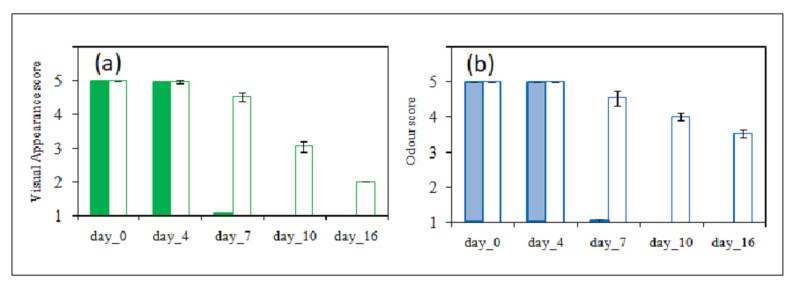
Visual appearance, (**a**), and odour score, (**b**), of samples of broccoli raab stored at 5 °C for 16 days in non-microperforated PET (NMP) and in microperforated PET (MP), for 16 days storage at 5 °C. Visual quality scale as reported in Figure 1. Odour scale: 5 = typical odour, no off-odor, 4 = slight typical odour, no off-odour, 3 = absence of typical odour, 2 = moderate off-odour, 1 = severe off-odour. A score of 3 was considered the limit of marketability. For NMP packaging, the bars are filled with colour, while for microperforated MP packaging, the bars are represented with an empty line (outline).

The composition of the internal atmosphere and the duration of storage significantly affected the appearance and odour of fresh-cut broccoli raab (Figure 4). Both packaging types showed a gradual decline in visual quality over time (Figure 4a); in NMP, excessive humidity due to water condensation, combined with high CO_2_ levels, likely contributed to tissue degradation, causing loss of firmness. Assuming a linear decrease in visual quality score between day 4 and day 7, samples stored in NMP reached the threshold of marketability after approximately 6 days. Conversely, samples in MP maintained marketability until day 10, when the samples appeared yellowish.

Odour evaluations carried out immediately after opening the packaging (Figure 4b) highlighted that NMP samples developed off-odours by day 7, which panellists perceived as unacceptable. The anaerobic conditions are likely the reason for the formation of unpleasant volatile compounds inside the NMP packaging. As for the visual quality, assuming a linear decline in odour score from day 4 to day 7, the marketability threshold for NMP samples was estimated to be approximately 6 days. In contrast, MP samples retained acceptable odour quality throughout the 16-day storage period, despite a gradual decline. No off-odours were then perceived in MP samples during all the storage periods.

This is consistent with previous findings by [18], who reported an increase in off-odour scores in *Brassica* spp. stored at 5 °C in low OTR PP film. In that study, a rapid drop in the percentage of oxygen to an approximate value of 2% was observed within 2 days of storage, negatively affecting sensory perception.

### 3.3. VOC Fingerprint of Fresh-like Broccoli Raab

The evolution of the VOC fingerprint of broccoli raab was monitored throughout storage using non-invasive in-packing headspace sampling. This approach minimised tissue disruption and the associated enzymatic artefacts, allowing the detection of volatiles representative of aroma likely perceived by consumers. In contrast, sample preparation by homogenisation, as reported by [18], results in complete disruption of plant tissues and may mimic the tissue degradation that occurs within the oral cavity, during mastication. Moreover, the SPME conditioning and extraction steps, typically lasting over 40 min at a minimum of 40 °C, may lead to the degradation of volatile compounds or attenuate the differences between treatments. Instead, the procedure used in this study avoided disturbing the sample and was performed at 5 °C, allowing the detection of volatile compounds present in the sample headspace under refrigerated conditions. Table 1 summarises information on the VOCs identified, including the chemical family, their retention time, peak area percentage, and odour description.

Method reproducibility, accounting for SPME, GC-MS, and sample variability, was expressed as RSD% of the analyte peak area and ranged from 15 to 25%. Thirteen VOCs were detected in initially packaged broccoli raab, analysed 1 h after packaging, including 4 sulphur-containing compounds, 3 carbonyl compounds, 2 terpenes, and 4 nitriles. The total VOC peak area in NMP was 1.5 times greater than in MP, suggesting a faster development of volatiles in NMP. Among VOCs, methyl thiocyanate was found only in MP, and dimethyl sulphide (DMS) and 2-methylbutanenitrile exclusively in NMP (Table 1). DMS was present already after 1 h in broccoli raab packaged in NMP; meanwhile, dimethyl disulfide (DMDS) was present in both samples; however, in NMP, DMDS peak area was the most abundant within the investigated compounds (47%). Similarly, [20] reported a high percentage of total sulphur compounds (83%) already on day 0 of storage. Previous studies have linked high levels of sulphur volatiles to methionine and cysteine [22]; similarly, degradation of the free amino acid named L-S-methylcystein sulphide produced DMS and DMDS [23].

**Figure 5:**
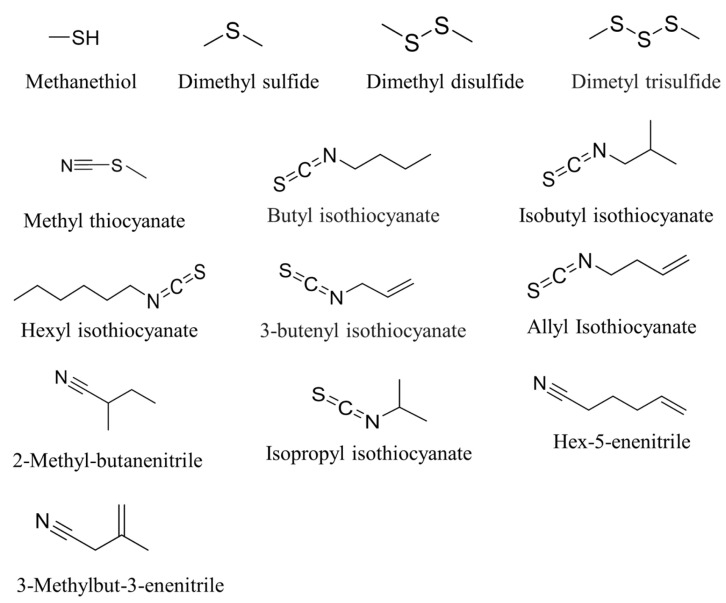
Structures of the main detected sulphurous and nitrile compounds found in fresh-cut broccoli raab during storage at 5 °C.

Given the extremely low odour threshold of these compounds, detectable by the human nose at concentrations as low as one part per trillion [24], their role in the characteristic flavour profile of *Brassica* vegetables is likely significant, even before off-odours are perceptible. Additionally, volatile profiles of both NMP and MP initial samples were characterised by the presence of isothiocyanates (butyl isothiocyanate and 3-butenyl isothiocyanate) [25,26], and nitriles (3-methylbut-3-enenitrile and hex-5-enenitrile). Both isothiocyanates and nitriles are produced during enzymatic hydrolysis of glucosinolates, formed naturally against plant pathogens, in cruciferous vegetables, as a result of plant tissue disruption [27]. These compounds have been the subject of intensive research due to their beneficial effects on human health. Isothiocyanates are also partially accountable for the specific pungent notes [28]. The structures of the main sulphurous and nitrile compounds detected in the present work are illustrated in Figure 5.

One hour after packaging, the MP sample volatile profile was characterised by the presence of C6 compounds, Z-3-hexen-1-ol acetate and Z-3-hexen-1-ol, which are known to derive from the lipoxygenase pathway from fatty acids and are responsible for the green-leaf flavour [29]. The peak area of these compounds took into account 26% and 15% of the total VOCs peak area detected in MP, compared to 3.0% and 3.5% in NMP, respectively. Terpenes such as limonene and caryophyllene were also detected. Limonene, generated as a plant defence mechanism from terpenoids [29], is known to confer floral notes to vegetables, likely giving an accountable contribution to the fresh odour, both in MP and in NMP.

(1R,4E,9S)-4,11,11-trimethyl-8-methylidenebicyclo[7.2.0]undec-4-ene, better known as ß-caryophyllene, has been reported in the essential oils of several plants [30] characterised by spicy notes, including *Brassica juncea* essential oil and oleoresins [31]. It has been found to have various beneficial properties, such as anticancer, antioxidant, and antimicrobial [32], anticonvulsant, analgesic, myorelaxing, sedative, and antidepressant effects [33]. Both terpenes were previously reported in Brassicaceae species [34]. Also, acetic acid was found in MP and NMP, with a peak area percentage of 20 and 8%, respectively.

From the results discussed above, it looks like the packaging has already started playing a role in affecting the VOC profile from the earliest stages of storage (1 h after packaging), although no sensory difference was appreciated.

### 3.4. Evolution of Broccoli Raab VOCs During Storage as Affected by Packaging Conditions

**Figure 6:**
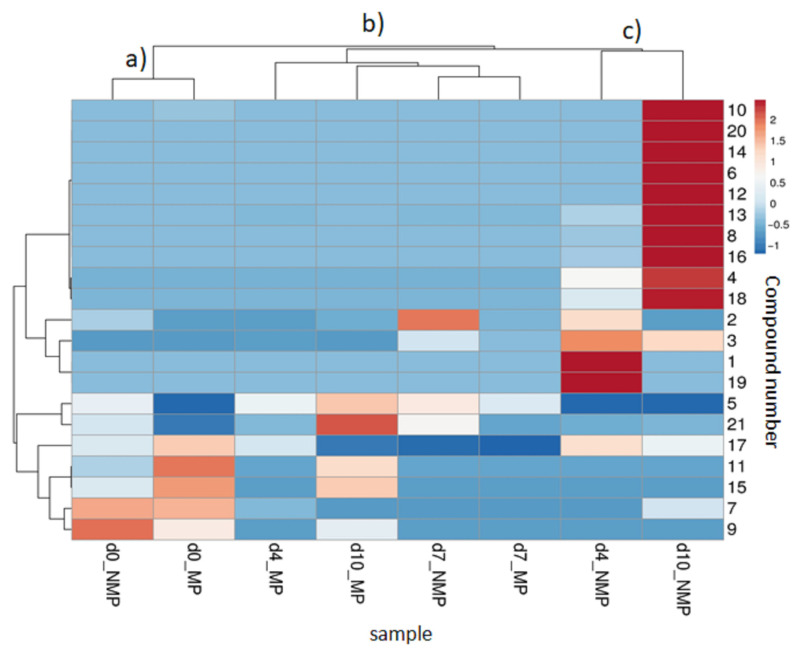
Hierarchical clustering heatmap of broccoli raab VOCs during storage at 5 °C of fresh-cut broccoli raab stored at 5 °C in non-microperforated PET (NMP) and in microperforated PET (MP). d0, 1 h storage; d4, four days storage; d7, seven days storage; d10, ten days storage; a, cluster of initial samples (d0_MP, d0_NMP); b, cluster of MP samples at days 4, 7, and 10 (d4_MP, d7_MP, d10_MP) and NMP sample at day 7 (d7_NMP); c, cluster of NMP samples at days 4 and 10 (d4_NMP, d10_NMP). The compound numbers on the right y-axis correspond to the VOCs listed in Table 1.

During the storage, eight compounds not present in fresh samples were detected (Table 1), six of which were sulphur-containing and found exclusively in NMP. On day 10, the total VOC peak area in NMP was 750 times higher than in MP, suggesting extensive volatile accumulation in packaging under anaerobic conditions. A Hierarchical Cluster Analysis with a heatmap colour representation (Figure 6) was performed to explore the VOC profile evolution of MP and NMP samples.

The VOC fingerprints of fresh-like and packaged samples were used to assess the impact of packaging material and perforation on headspace VOC profiles during storage. Samples were grouped into three main clusters: a, initial samples (d0_MP, d0_NMP); b, MP samples at days 4, 7, and 10 (d4_MP, d7_MP, d10_MP) and NMP sample at day 7 (d7_NMP); c, NMP samples at days 4 and 10 (d4_NMP, d10_NMP).

As discussed above, results showed that 1 h storage was already sufficient to cause small differences in the VOC profile. Indeed, as shown in Figure 6, at day 0, d0_NMP showed higher levels of 3-methylbut-3-enenitrile (compound **9**), and d0_MP showed higher amounts of acetic acid (compound **17**) and of the “green” notes Z-3-hexen-1-ol acetate and hexen-1-ol (compounds **11**, **15**). With time, as expected, VOCs underwent a wide variety of modifications. In MP samples, by day 4 of storage, most VOCs decreased without new compound formation, suggesting no olfactory defect, consistently with sensory scores; this decrease, likely due to a low total amount of odorants, was not perceived by panellists (Figure 4). In contrast, a new group of VOCs, including methanethiol, allyl isothiocyanate derivative, and ethanol (compounds **1**, **19**, **3**) was detected in NMP packaging, placing d4_NMP far from the cluster “a” in the heatmap (Figure 6). Methanethiol has been previously reported as a key metabolite responsible for off-odours in broccoli stored under controlled atmosphere containing 0.5% oxygen [35] and in the headspace of broccoli seedlings stored under anaerobiosis [22]. Methanethiol is also considered a precursor of dimethyl disulfide and trisulfide [19].

Compound **19** was attributed to an allyl isothiocyanate derivative for the characteristic ions of *m*/*z* 99 and 72; however, no specific structure could be assigned. Allyl isothiocyanate is among the most extensively studied isothiocyanates, with numerous reports demonstrating its anticancer activity in both cultured cancer cells and animal models. In addition, it exhibits high bioavailability, with nearly 90% of the orally administered dose being absorbed [36]. The presence of ethanol suggested the occurrence of fermentative processes due to the anaerobic conditions (Figure 2), as previously observed [37]. However, the contribution of microbial fermentation to the observed VOCs was not directly assessed in this study. At this stage, these compounds were not perceived as unpleasant odours by the panellists, as shown in the overall odour balance of the sample (Figure 4).

By day 7, MP broccoli raab showed a further loss of VOCs, without generating any new volatile compounds. In contrast, NMP broccoli raab were characterised by the strong presence of DMS (compound **2**). MP samples received the odour score of 4.5, associated with a slight change in characteristic odour; whereas NMP received the odour score of 1, indicative of the presence of off-flavour.

On day 10, MP VOCs were characterised by a major presence of 2-methylbutanenitrile, caryophyllene, Z-3-hexen-1-ol acetate, and hexen-1-ol (compounds **5**, **21**, **11**, **15**), likely due to enzymatic degradation from tissue senescence. None of these compounds introduced off-odours, as suggested by the sensory analysis, scoring 4. In contrast, NMP samples exhibited extensive accumulation of sulphur-containing compounds (peaks **10**, **20**, **14**, **6**, **12**, **13**, **8**, **16**, **4**, **18**), including sulphides, isothiocyanates, and nitriles. The anaerobic atmosphere caused evident tissue damage, as described in Section 3.2, which altered the volatile fingerprint. Since VOCs were measured directly in the headspace without sample homogenisation, their profile was effectively influenced by tissue integrity, allowing the detection of differences between samples that would likely have been masked or attenuated by sample homogenisation. In the more extensively damaged NMP tissues, enzymes and their precursors, normally compartmentalised within the cells, came into contact, facilitating enzymatic reactions that produced sulphur-containing volatiles. This mechanism, combined with non-enzymatic reactions under anaerobic conditions, contributed to the strong, unpleasant notes perceived by the panellists (score 1). Overall, degradation reactions yielding VOCs occurred to a lower extent in MP due to the higher O_2_, whereas anaerobic conditions in NMP enhanced the degradation rate of cell structures and volatile formation. By day 16, both samples were judged unacceptable, NMP due to off odour and MP due to discoloration, thus VOCs were not analysed further. Correlations between VOCs and odour scores were confirmed by PCA (Supplementary Materials).

## 4. Conclusions

Microperforated PET packaging effectively mitigated degradation processes in fresh-cut broccoli raab and extended its marketable shelf-life up to 10 days under refrigerated storage at 5 °C, compared to 6 days in non-microperforated packaging. For MP, the main factor limiting marketability was the progressive decline in visual quality, whereas in NMP, both the development of off-odours and deterioration in visual quality significantly compromised product acceptability. VOCs analysis, performed directly from the packaging headspace using in-packing SPME to avoid tissue disruption, demonstrated that packaging type influenced the volatile profile as early as one hour after storage onset, although these early changes did not result in perceptible odour differences. MP packaging delayed the accumulation of some sulphur-containing compounds and preserved green-leaf volatiles, whereas NMP samples rapidly accumulated DMDS, DMS, methanethiol, and other compounds within 4–7 days and showed strong unpleasant sensory notes. Despite these promising outcomes, the optimal number and diameter of perforations require further investigation to balance gas exchange and prevent premature visual senescence. Additionally, the impact of temperature fluctuations on VOC formation, gas composition, and shelf-life should be explored. Overall, these findings highlight the potential of microperforation even when using PET-based materials and support its application as a viable strategy for developing environmentally sustainable solutions in fresh-cut produce storage.

## Figures and Tables

**Table 1:** Fingerprint VOCs of fresh-cut broccoli raab. Peak area percentage of Volatile Organic Compounds found in broccoli raab headspace after 0, 4, 7, and 10 days of storage at 5 °C in non-microperforated PET (NMP) and in microperforated PET (MP).

Compound Number	RI	IUPAC Name	Common Name	d0-NMP (%)	d0-MP (%)	d4-NMP (%)	d4-MP (%)	d7-NMP (%)	d7-MP (%)	d10-NMP (%)	d10-MP (%)	Odour
*Sulphurous organic compounds*												
**1**	600	Methanethiol		-	-	3.0 ± 0.3	-	-	-	-	-	Sulphurous
**2**	601	Methylsulfanylmethane	Dimethyl sulphide	4.4 ± 0.5	-	0.10 ± 0.6	-	14.7 ± 0.5	3.9 ± 0.4	-	1.5 ± 0.5	Sulphurous
**4**	1001	(Methyldisulfanyl)methane	Dimethyl disulphide	46.6 ± 0.6	8.2 ± 0.4	60.1 ± 0.4	5.6 ± 0.5	27.1 ± 0.4	12.3 ± 0.4	37.3 ± 0.5	27.0 ± 0.4	Sulphurous
**6**	1198	2-Isothiocyanatopropane	Isopropyl isothiocyanate	-	-	-	-	-	-	0.4 ± 0.6	-	Alliaceous
**8**	1201	1-Isothiocyanatobutane	Butyl isothiocyanate	2.8 ± 0.6	3.3 ± 0.5	2. 7 ± 0.3	-	-	-	15.3 ± 0.6	-	Sulphurous
**12**	1303	1-Isothiocyanato-2-methylepropane	Isobutyl isothiocyanate	-	-	-	-	-	-		-	Green
**14**	1305	3-Isothiocyanatoprop-1-ene −	Allyl Isothiocyanate	-	-	-	-	-	-		-	Sulphurous
**16**	1400	(Methyltrisulfanyl)methane	Dimethyl trisulphide	-	-	8.0 ± 0.5	-	-	-	34.2 ± 0.5	-	Alliaceous
**18**	1402	4-Isothiocyanatobut-1-ene	3-Butenyl isothiocyanate	2.4 ± 0.5	5.6 ± 0.5	9.7 ± 0.3	-	-	-	11.6 ± 0.5	-	Aromatic Pungent
**19**	1501		Unknown—Allyl Isothiocyanate derivative	-	-	14.7 ± 0.3	-	-	-	-	-	Sulphurous
**20**	1600	1-Isothiocyanatohexane	Hexyl isothiocyanate	-	-	-	-	-	-		-	Sharp green
*Alcohols, acids, and esters*												
**3**	603	Ethanol		-	-	1.4 ± 0.4	9.7 ± 0.5	42.8 ± 0.4	58.5 ± 0.5	0.3 ± 0.5	-	Alcoholic
**11**	1302	[(Z)-2,5-Dimethylhex-3-enyl] acetate oppure (E)	*cis*-3-Hexenyl acetate	3.0 ± 0.3	26.1 ± 0.5	-	-	-	-	-	14.5 ± 0.6	Green fruity
**15**	1398	(Z)-Hex-3-en-1-ol	Leaf alcohol	3.5 ± 0.3	15.2 ± 0.5	-	-	-	-	-	10.3 ± 0.4	Green
**17**	1401	Acetic acid	Ethanoic acid	8.4 ± 0.3	20.2 ± 0.4	0.08 ± 0.3	32.3 ± 0.4	2.2 ± 0.5	6.1 ± 0.4		4.7 ± 0.4	Aacidic
*Terpenes*												
**7**	1200	1-Methyl-4-prop-1-en-2-cylcyclohexene	Limonene	9.4 ± 0.4	14.1 ± 0.5	-	5.1 ± 0.5	-	-	-	-	Citrus
**21**	1600	(1*R*,4*E*,9*S*)-4,11,11-Trimethyl-8 methylidenebicyclo [7.2.0]undec-4-ene	ß-Caryophyllene	8.6 ± 0.4	2.4 ± 0.5	-	21.8 ± 0.5	8.0 ± 0.4	8.4 ± 0.5	-	26.7 ± 0.5	Spicy
*Nitriles*												
**5**	1100	Cyclobutane-1-carbonitrile	2-Methyl-butanenitrile	6.1 ± 0.5	-	-	25.5 ± 0.5	5.2 ± 0.3	10.9 ± 0.3	-	12.3 ± 0.5	Sweet, musty
**9**	1202	3-Methylbut-3-enenitrile	Methallyl cyanide	1.42 ± 0.6	1.3 ± 0.5	-	-	-	-	-	0.7 ± 0.5	Fruity
**10**	1300	Methyl thiocyanate	Methyl sulfocyanate	-	0.7 ± 0.4	-	-	-	-	-	-	Sulphury onion
**13**	1304	Hex-5-enenitrile	5-Cyano-1-pentene	3.4 ± 0.3	2.8 ± 0.6	0.3 ± 0.3		-	-	0.9 ± 0.5	2.0 ± 0.5	-

## Data Availability

The data presented in this study are available on request from the corresponding author due to privacy and legal requirements by the project founder.

## Supplementary Materials

The following supporting information can be downloaded at: https://www.mdpi.com/article/10.3390/foods14244283/s1.

## Abbreviations

The following abbreviations are used in this manuscript:

PET	Polyethylene terephthalate
VOCs	Volatile organic compounds
MP	Microperforated
NMP	Non-microperforated
PP/PA	Polypropylene/Polyamide
PP/PET	Polypropylene/Polyethylene terephthalate
DMDS	Dimethyl disulphide
DMTS	Dimethyl trisulphide
WL	Weight loss
SPME	Solid phase micro-extraction
OTR	Oxygen Transmission Rate
