# Supplementary figures and images for: Headspace Volatile Profile of Fresh-Cut Broccoli Raab in PET Packaging as Affected by Microperforation
Source: Foods. 2025 Dec 12;14(24):4283. doi: 10.3390/foods14244283 (PMC12733288; doi:10.3390/foods14244283)

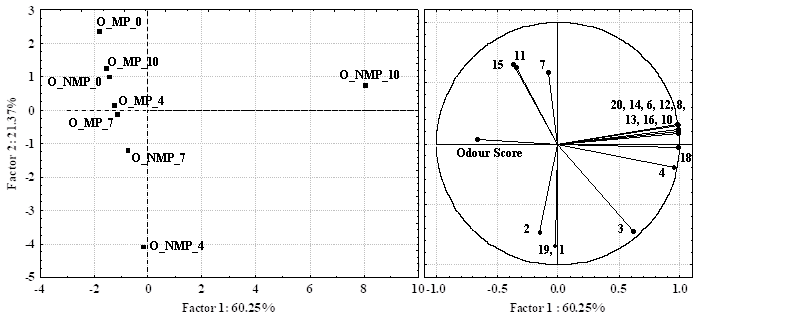

Supplement: Supplementary file 1 [file foods-14-04283-s001.zip › foods-3963793-supplementary.tif]
